# First reported enterovirus D68 infection in pediatric patients from the Caribbean region: evidence of spread from the U.S. outbreak

**DOI:** 10.26633/RPSP.2017.11

**Published:** 2017-02-08

**Authors:** SueMin Nathaniel, Shalauddin Ahmed, Jennifer Wilson, Cristina Gutierrez, Dave D. Chadee, Babatunde Olowokure, Pablo M. de Salazar

**Affiliations:** 1 Caribbean Public Health Agency Port of Spain Trinidad and Tobago Caribbean Public Health Agency, Port of Spain, Trinidad and Tobago.; 2 Ministry of Health and the Environment Roseau Commonwealth of Dominica Ministry of Health and the Environment, Roseau, Commonwealth of Dominica.; 3 Ministry of Health Seniors and Environment Hamilton Bermuda Ministry of Health, Seniors and Environment, Hamilton, Bermuda.; 4 University of the West Indies St. Augustine Trinidad and Tobago University of the West Indies, St. Augustine, Trinidad and Tobago.

**Keywords:** Enterovirus D, human, enterovirus infections, Caribbean region, Dominica, Bermuda, Enterovirus humano D, infecciones por enterovirus, Región del Caribe, Dominica, Bermuda

## Abstract

The 2014 enterovirus D68 (EV-D68) outbreak in the United States raised concerns about the introduction of the virus in the Caribbean region. The objective of this study was to provide rapid evidence of the introduction of EV-D68 strains in the Caribbean region during the 2014 outbreak in the United States, using a relatively simple phylogenetic approach. From October 2014 to May 2015, four EV-D68 cases from two countries (Bermuda and Dominica) were detected at the regional referral laboratory at the Caribbean Public Health Agency (Port of Spain, Trinidad and Tobago) based on molecular testing of respiratory specimens. All cases were children presenting to hospitals with moderate respiratory distress. No cases of acute flaccid paralysis were detected. Phylogenetic analysis of the Caribbean strains showed more than 99% similarity with the 2014 U.S.-outbreak strain, providing evidence of the introduction and circulation of the virus in the region.

## RESUMEN

El brote de enterovirus D68 (EV-D68) registrado en el 2014 en los Estados Unidos suscitó preocupación acerca de la introducción del virus en el Caribe. El objetivo de este estudio fue aportar pruebas rápidas, mediante la adopción de un enfoque filogénico relativamente sencillo, de que durante ese brote ingresaron en el Caribe cepas del EV-D68. Entre octubre del 2014 y mayo del 2015, el laboratorio regional de referencia ubicado en el Organismo de Salud Pública del Caribe (Puerto España, Trinidad y Tabago) detectó cuatro casos de EV-D68 provenientes de dos países (Bermudas y Dominica) mediante el análisis molecular de muestras respiratorias. Todos los casos correspondían a niños que acudieron al hospital con dificultad respiratoria moderada. No se detectó ningún caso de parálisis flácida aguda. El análisis filogénico de las cepas encontradas en el Caribe demostró una semejanza superior al 99 % con la cepa responsable del brote del 2014 en los Estados Unidos, lo que demuestra la introducción y la circulación del virus en la región.

During the past few years, enterovirus D68 (EV-D68) has emerged as a major viral pathogen, with recent outbreaks occurring in the United States in 2014 (1). These outbreaks led to the enhancement of surveillance systems, which revealed EV-D68 outbreaks in Canada (2) and Chile (3), as well as in several other countries in Europe (4) and Asia (5). The reported EV-D68 infections were mostly associated with severe respiratory illness in children with underlying respiratory conditions such as asthma (1), but recently an association between infection with EV-D68-specific clades such as B1 and neurological illnesses was suggested (6).

## Surveillance for EV-D68 in the Caribbean

The recent increase in the number of cases prompted the referral laboratory at the Caribbean Public Health Agency (CARPHA) (Port of Spain, Trinidad and Tobago) to implement surveillance for EV-D68 as part of its acute respiratory infection (ARI) sentinel surveillance in the Caribbean region. The laboratory surveillance was carried out to determine the potential introduction and circulation of EV-D68 in the Caribbean region during the 2014 U.S. outbreak. As a part of a routine surveillance program in the English- and Dutch-speaking Caribbean, respiratory specimens (nasopharyngeal swabs and nasopharyngeal aspirates) from patients presenting to health care facilities with influenza-like illness (ILI) or ARI are sent to the regional referral laboratory (CARPHA). Samples are tested for influenza and non-influenza etiology including rhinovirus, parainfluenza, human metapneumonia virus, adenovirus, and respiratory syncytial virus. Since October 2014, samples received in the laboratory that tested negative for influenza were also tested for enterovirus (generic detection) using real-time reverse transcription PCR (RT-PCR). If a positive result was obtained, molecular testing for EV-D68 was performed based on a previously described procedure (7). The objective of the analysis was to provide rapid evidence of the introduction of EV-D68 strains in the Caribbean region during the 2014 outbreak in North America, using this rather simple phylogenetic approach. CARPHA’s research ethics committee authorized the publication of the study.

## Evidence and spread in pediatric patients

From a total of 259 samples received between October 2014 and May 2015, 14 samples tested positive using panenterovirus RT-PCR. EV-D68 was identified in four clinical specimens from two different countries (Bermuda and Dominica). Because pan-enterovirus RT-PCR can detect all members of the enterovirus genus, the remaining 10 samples were considered probable non-EV-D68 human enterovirus–producing respiratory infections. The specimens that tested positive for EV-D68 were obtained from two patients from Bermuda presenting with severe acute respiratory tract infections (SARIs) in mid-October, and two patients from Dominica presenting with SARIs at the end of November and beginning of December, respectively. The four positive samples had been collected within two days of symptom onset. All samples were negative for the additional respiratory virus panel.

The patients’ medical records were reviewed retrospectively to evaluate clinical manifestations at admission, diagnostics, management, and outcome as well as relevant medical and epidemiological history. Agreement from parents and guardians of the patients was obtained for publication. All patients were hospitalized children under 5 years old and presented at health facility emergency rooms with moderate to severe respiratory illness. Three of the patients reported recent travel. One of the two patients from Dominica had returned from the French overseas territory of Martinique three weeks before admission to the hospital. The two patients from Bermuda reported returning from the United States two and three weeks respectively before presenting to the hospital. No cluster involving family members or schoolmates was reported. History of reactive airway disease was recorded for two patients. Main reported manifestations at admission were difficulty of breathing and tachypnea, irritability, wheezing, and cough. Fever was reported or recorded in only two cases. Patients were diagnosed as presenting with lower respiratory tract infection based on clinical and radiological findings, and all received treatment with bronchodilators and supportive symptomatic treatment. Both patients from Dominica received antibiotics during hospitalization because of initial suspected bacterial etiology, but no positive bacterial cultures were reported. None of the patients required admission to the intensive care unit or required mechanical ventilation during admission. Three of the four patients improved and were discharged within 72 hours of admission. One of the two patients from Dominica, who was 8 months old, remained hospitalized for 10 days diagnosed with community-acquired pneumonia. This patient was eventually discharged on empirical oral antibiotic treatment. A summary of the four patients’ clinical, demographic, and epidemiologic features is shown in Table 1.

**TABLE 1:** Clinical, demographic, and epidemiologic features of enterovirus D68 (EV-D68) patients (*n* = 4), Caribbean region, October 2014–May 2015^a^

Patient no.	Country of origin	Sex	Age	Date of diagnosis	Travel history^b^	Symptoms at admission	Lung X-ray findings	Underlying conditions
1	Bermuda	Male	4 years	10/14/14	United States (3 weeks)	Cough, low-grade fever, shortness of breath, wheezing	Bilateral basal pneumonia	Reactive airway disease
2	Bermuda	Female	2 years	10/17/14	United States (2 weeks)	Abdominal pain, cough, difficulty of breathing, vomiting	No reported findings	Reactive airway disease
3	Dominica	Male	4 months	11/20/14	–^c^	Cough, difficulty of feeding, fever, irritability, nasal flaring, rhinorrhea	Bilateral patchy infiltrates in the bases	No
4	Dominica	Female	8 months	12/3/14	Martinique (3 weeks)	Difficulty of breathing, irritability, nasal flaring	Broncho-pneumonia	Intrauterine growth restriction Unspecified musculoskeletal disorder

***Source:*** Prepared by the authors based on the study results.

a Based on available clinical, demographic, and epidemiologic data obtained retrospectively from medical records.

b Countries/overseas regions visited and approximate time between return from travel and hospitalization.

c Not applicable.

**FIGURE 1.**
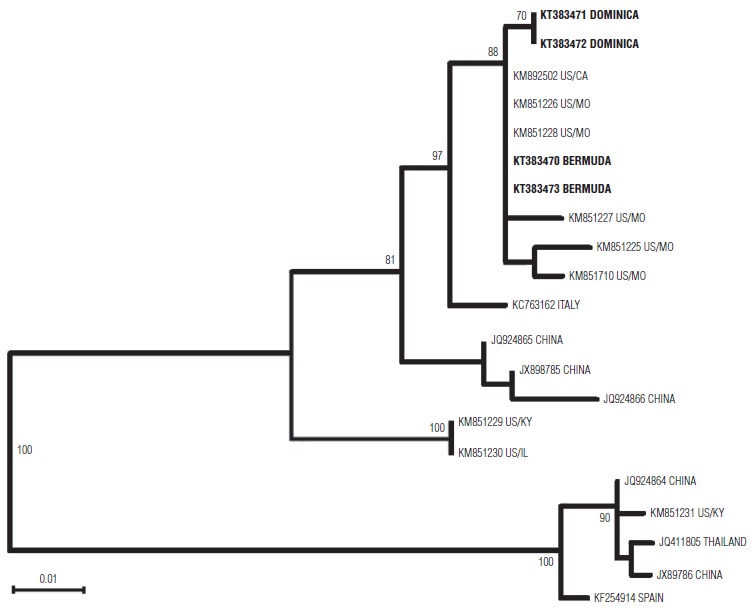
Phylogenetic analysis of enterovirus D68 (EV-D68) partial viral protein 1 (VP1) sequences, Caribbean region, 2014^a^

***Source***: Prepared by the authors based on the study results.

Samples were sent to the U.S. Centers for Disease Control and Prevention (CDC) (Atlanta, Georgia). EV-D68 was confirmed in all four clinical samples by real-time RT-PCR (http://www.fda.gov/downloads/MedicalDevices/Safety/EmergencySituations/UCM446784.pdf). Partial sequencing of the 3′ half of the genomic region encoding the viral protein 1 (VP1) domain of the four EV-D68-positive clinical specimens was performed as previously described (7, 8). Sequences were aligned with published EV-D68 sequences downloaded from GenBank (July 2015) (http://www.ncbi.nlm.nih.gov/genbank). Phylogenetic analysis was carried out using the neighbor-joining unweighted pair group method with arithmetic mean (UPGMA); maximum likelihood (ML); and minimum evolution constructs (Molecular Evolutionary Genetics Analysis (MEGA) 6.0 software (Center for Evolutionary Medicine and Informatics, The Biodesign Institute, Tempe, Arizona, United States)). As all analysis showed similar results, only the neighbor-joining tree constructed with 1 000 bootstrap replications in the tree ligand is presented here (Figure 1). Study sequences were deposited in the GenBank database under accession numbers KT383470, KT383471, KT383472, and KT383473. Comparison of the study strains and available reference sequences showed that the Bermudian strains were a 100% match and shared > 99% nucleotide identity with the virus circulating in the United States in 2014. The two Dominican strains were also a 100% match and showed 99% nucleotide identity with both the Bermudian strains and the circulating virus from the United States (Figure 1). According to the phylogenetic tree, the four isolates were grouped within the B1 clade (6), which has been recently related with potential neurological illness. However, none of the cases in the cohort showed neurological manifestations during the six-month post–acute episode follow-up.

## Limitations

As the objective of this study was to provide rapid evidence of the introduction of EV-D68 strains in the Caribbean region during the 2014 outbreak in North America, a rather simple phylogenetic approach was used. Therefore, the phylogenetic analysis had some limitations, including the small number of study strains, the relatively short length of the sequences, and the intrinsic limitations of the tree construction criterion. Other limitations of this study were the retrospective medical assessment and the small number of clinical cases identified. Because EV-D68 epidemiology has developed over a few decades from sporadic cases to worldwide cluster occurrence, strong analysis of the evolutionary status by phylodynamic approach could be invaluable for understanding changes in viral transmission, such as increased infectivity, replication potency, or host immune evasion, as well as for development of vaccines and antiviral treatment strategies.

## Conclusions

This phylogenetic analysis provides evidence of the introduction and circulation of EV-D68 in the Caribbean region during the 2014 outbreak in the United States. Genetic analysis of the infections of the four confirmed cases shows the identified strains are strongly related to those reported in the U.S. 2014 outbreak. While the virus has only been detected in two Caribbean countries (Bermuda and Dominica), cases in other countries in the region are likely due to the high volume of travel and trade with North America and other parts of the world. Therefore, appropriate surveillance strategies need to be implemented in the region. Clinicians should consider EV-D68 in the differential diagnosis when patients present with severe acute respiratory illness, especially in infants and children with history of asthma. The importance of maintaining coordinated regional surveillance and awareness is essential for ensuring an adequate public health response.

### Acknowledgments.

The authors thank Shannon L. Rogers and the Polio and Picornavirus Laboratory Branch, U.S. Centers for Disease Control and Prevention (CDC) (Atlanta, Georgia, United States), for assistance with testing confirmation and sequencing.

### Disclaimer.

Authors hold sole responsibility for the views expressed in the manuscript, which may not necessarily reflect the opinion or policy of the Caribbean Public Health Agency (Port of Spain, Trinidad and Tobago); the Ministry of Health, Seniors and Environment of Bermuda (Hamilton); the Ministry of Health and the Environment, Commonwealth of Dominica (Roseau); the *RPSP/PAJPH*; or the Pan American Health Organization (PAHO).

## Footnotes

a Phylogenetic tree constructed by neighbor-joining analysis, with 1 000 bootstrap replications (BRs) in the tree ligand, using Molecular Evolutionary Genetics Analysis (MEGA) 6.0 software (Center for Evolutionary Medicine and Informatics, The Biodesign Institute, Tempe, Arizona, United States). Only BRs 70% and higher are shown. Branch lengths are drawn to scale. Scale bar indicates 0.01 substitutions per site. Available reference sequences were downloaded from GenBank (July 2015) (http://www.ncbi.nlm.nih.gov/genbank). Sequences from this study, and reference sequences, are identified by accession number and country of origin. Study sequences (in boldface) were found closely clustered to the U.S. 2014 strains (KM892502, KM851225–KM851228, and KM851710).
